# Advances in research regarding epithelial-mesenchymal transition and prostate cancer

**DOI:** 10.3389/fcell.2025.1583255

**Published:** 2025-05-30

**Authors:** Xi Wei, Rui Liu, Wei Li, Qi Yu, Qing Tao Yang, Tao Li

**Affiliations:** Department of Urology, The Affiliated Hospital of Guizhou Medical University, Guiyang, China

**Keywords:** prostate cancer, epithelial-mesenchymal transition, tumor progression, tumor drug resistance, heterogeneity

## Abstract

Prostate cancer (PCa) is the most prevalent cancer in men and the fifth leading cause of cancer-related mortality among men globally. Despite substantial advancements in patient prognosis attributable to improvements in PCa treatment, individuals with metastatic castration-resistant prostate cancer continue to experience poor outcomes. Epithelial-mesenchymal transition (EMT) is characterized as a cellular event in which epithelial cells adopt a mesenchymal phenotype while simultaneously losing their epithelial characteristics. EMT has been demonstrated to be associated with the progression of PCa, encompassing tumor metastasis, recurrence, drug resistance, and the development of an immunosuppressive microenvironment. Consequently, this review synthesizes recent studies on EMT in PCa, consolidating the events mediated by EMT in the progression of PCa and the molecular mechanisms linked to EMT activation in this context.

## Introduction

PCa is the most prevalent malignant tumor of the male genitourinary system, ranking second in incidence and fifth in mortality among malignant tumors in men globally (Siegel et al., 2023). Treatment strategies for PCa typically depend on factors including prostate-specific antigen levels, pathological type, Gleason score, and clinical stage (Teo et al., 2019; Gillessen et al., 2023). For early-stage (T1 and T2) PCa, favorable outcomes can be attained through active surveillance, local radiotherapy, or radical prostatectomy (Teo et al., 2019; Gillessen et al., 2023). However, due to the extended latent period of PCa, approximately 70% of PCa cases are either locally advanced or widely metastatic at the time of diagnosis. Treatment for advanced PCa primarily relies on androgen deprivation therapy, radiochemotherapy, and targeted therapies (Falagario et al., 2023; Preisser et al., 2024). Although these treatments initially yield favorable results, resistance inevitably develops, resulting in the progression to castration-resistant prostate cancer (CRPC). Once CRPC manifests, current drugs and treatment methods frequently fail to yield effective outcomes (Falagario et al., 2023; Preisser et al., 2024). Notably, mounting evidence suggests that EMT plays a crucial role in the progression of PCa, encompassing recurrence, drug resistance, metastasis, and the development of CRPC (Dunning et al., 2011; Teng et al., 2021). Thus, understanding the cellular and molecular mechanisms, including EMT, involved in PCa progression could offer potential therapeutic strategies to mitigate PCa-related mortality.

EMT is recognized as a fundamental mechanism in cancer metastasis (Haffner et al., 2021; Cha et al., 2020). Emerging evidence indicates that EMT is not only closely associated with tumor metastasis but also with tumor stemness, cytokine release, cancer-associated angiogenesis, immune evasion, and chemoresistance (Pan et al., 2021; Lambert and Weinberg, 2021). Consequently, EMT is widely acknowledged as a hallmark of cancer and is considered a potential contributor to mortality in most solid tumors, including PCa. Notably, extensive evidence supports that EMT is associated not only with the metastatic and disseminative capabilities of PCa but also with the development of CRPC. It has been reported to exhibit significant cross-talk with the androgen receptor (AR) signaling pathway (Dunning et al., 2011; Teng et al., 2021). Recent advances in single-cell RNA sequencing (scRNA-seq) have shed new light on the biological role of EMT in PCa (He et al., 2024). Analyses of metastatic PCa single-cell atlases have identified four functional EMT subtypes: classical mesenchymal, inflammatory, metabolic adaptive, and stem cell-like (He et al., 2024; Jia et al., 2025). Notably, the stem cell-like subtype (CD44^+^) is highly enriched in CRPC and exhibits significantly reduced sensitivity to androgen receptor (AR) inhibitors (He et al., 2024; Jia et al., 2025; Baumeister et al., 2021). While single-cell sequencing offers novel insights into EMT heterogeneity, its clinical translation remains challenging. A systematic exploration of EMT’s core mechanisms, regulatory networks, and heterogeneity—integrated with spatial transcriptomic approaches—will advance our understanding of PCa progression and provide critical theoretical foundations for optimizing precision therapies and risk stratification.

## Overview of EMT

EMT is defined as a cellular event in which epithelial cells acquire a mesenchymal phenotype while losing their epithelial characteristics (Haffner et al., 2021). This process is frequently observed in embryonic development, wound healing, organ fibrosis, and tumor metastasis (Cha et al., 2020). EMT, in conjunction with mesenchymal-epithelial transition (MET), plays a critical role in both early and late stages of embryonic development, including implantation, gastrulation, and heart formation (Pan et al., 2021). EMT is linked to various tumor functions, including tumor initiation, malignant progression, tumor stemness, cell migration, metastasis, and treatment resistance, and is often excessively activated in cancer cells (Haffner et al., 2021; Cha et al., 2020; Pan et al., 2021; Lambert and Weinberg, 2021). The hallmark characteristics of EMT involve epithelial cells gradually losing adhesion molecules such as E-cadherin and β-catenin, along with tight junction proteins. This process is accompanied by the expression of mesenchymal markers, including N-cadherin, R-cadherin, and vimentin, as well as increased levels of extracellular matrix and focal adhesion proteins (Pastushenko and Blanpain, 2019; Akhmetkaliyev et al., 2023). When tumor epithelial cells undergo EMT, their cell polarity and adhesion capabilities diminish, and they acquire a mesenchymal cell phenotype that promotes metastasis and drug resistance (Akhmetkaliyev et al., 2023). EMT is regulated by a series of transcription factors, primarily comprising Snail transcription factors, basic Helix-loop-helix (bHLH) proteins, and Zinc finger E-box binding homeobox (ZEB) transcription factors (Singh et al., 2018). EMT is also reported to be associated with, and regulated by, various oncogenic pathways, including Transforming Growth Factor-β (TGF-β), Wnt, and Notch signaling pathways. These pathways activate the aforementioned transcription factors, inhibiting the expression of E-cadherin and promoting the acquisition of a mesenchymal phenotype in tumor cells (Saitoh, 2023).

## EMT and progression in PCa

### EMT and metastasis in PCa

Since metastasis is the leading cause of cancer-related deaths in malignant tumors, developing targeted therapeutic strategies to inhibit cancer metastasis is crucial (Valastyan and Weinberg, 2011). EMT is typically associated with tumor metastasis and is recognized as a primary driver of this process. Mechanistically, following the EMT, epithelial cells acquire distinct mesenchymal characteristics (such as diminished cell polarity and adhesion), enabling them to invade the extracellular matrix as individual cells, thus marking the initial stage of tumor metastasis (Pastushenko and Blanpain, 2019; Akhmetkaliyev et al., 2023). Subsequently, these cells detach further from the primary tumor, enter the bloodstream, and colonize distant tissues, proliferating and initiating metastasis, indicating that tumor metastasis relies on the EMT process (Pastushenko and Blanpain, 2019; Akhmetkaliyev et al., 2023; Singh et al., 2018; Saitoh, 2023; Valastyan and Weinberg, 2011; Lu et al., 2022). This phenomenon has been observed in various tumors, including PCa. Previous studies have demonstrated that circulating PCa cells obtained from the blood of PCa patients exhibit upregulated expression of EMT-related genes (Pal et al., 2015), and these circulating tumor cells display a heightened mesenchymal phenotype (Pal et al., 2015). Similarly, clinical specimens from PCa patients have confirmed that metastatic tissues exhibit elevated levels of EMT-related genes and a more pronounced mesenchymal phenotype compared to primary tissues (Parol et al., 2021).

### EMT and metabolic traits in PCa

During EMT, PCa cells undergo marked metabolic reprogramming, endowing mesenchymal-like cells with unique adaptations that enhance survival and drive therapeutic resistance (Du et al., 2022). Recent studies reveal that mesenchymal-like PCa cells rely on glycolysis via the Warburg effect (Fontana et al., 2023), with single-cell metabolomics demonstrating a twofold increase in glycolytic flux compared to epithelial cells (Zhang Z. et al., 2023). Key regulators include HIF-1α-driven overexpression of GLUT1 and LDHA, alongside elevated PKM2 dimers that boost lactate production (Icard et al., 2022). Lactate excretion lowers tumor microenvironment pH, sustaining mesenchymal phenotypes through TGF-β signaling while suppressing CD8^+^ T cell activity to promote immune evasion (Kao et al., 2022). These cells also exhibit heightened glutamine dependence, with GLS1 expression increasing over fourfold (Du et al., 2022). Glutamine breakdown generates α-ketoglutarate (α-KG) to replenish the TCA cycle (Du and Hu, 2021), and ZEB1 reinforces this process by repressing miR-203, forming a positive feedback loop that upregulates GLS1 (Zhao et al., 2020). Glutamine-derived purines further support DNA repair under chemotherapy-induced stress, enhancing cell survival (Zhou et al., 2020). Mesenchymal-like PCa cells coordinately enhance fatty acid oxidation (FAO) and synthesis (FAS) (Tan et al., 2023). Single-cell lipidomics show upregulated CPT1A, which shuttles fatty acids into mitochondria for β-oxidation (Meng et al., 2024), while ACC phosphorylation inactivates FAS to relieve FAO suppression (Carroll et al., 2020). Concurrently, SREBP1-mediated lipid synthase expression increases membrane fluidity to facilitate migration (Du et al., 2022).

### Epigenetic effects of EMT on PCa

Emerging studies highlight the pivotal role of epigenetic modifications in PCa metastasis and therapy resistance by dynamically regulating EMT plasticity (Tan et al., 2022). The epigenetic network—comprising DNA methylation, histone modifications, and non-coding RNAs—not only shapes EMT heterogeneity but also drives adaptive evolution under therapeutic pressure (Tan et al., 2022). Whole-genome methylation analyses reveal increased hypermethylation at the CDH1 promoter in metastatic castration-resistant PCa (mCRPC), resulting in epithelial marker loss and ZEB1-mediated mesenchymal activation (Lee et al., 2020). Intriguingly, intermediate EMT (E/M) cells exhibit unique “bivalent domain” methylation patterns: partial methylation at epithelial gene promoters (FOXA1) and hypomethylation at mesenchymal loci (SNAI2), enabling rapid microenvironmental adaptation (Bhat et al., 2021; Liu Y. et al., 2023). Targeting DNMT3B reverses EMT phenotypes and restores drug sensitivity in enzalutamide-resistant cell lines (Wu et al., 2020). Histone modifications further regulate EMT transcription via chromatin remodeling (Yang et al., 2023). In AR inhibitor-resistant cells, H3K27ac enrichment at TWIST1 enhancers sustains mesenchymal traits (Chen et al., 2022), while dynamic H3K4me3 modifications at stemness-related genes (SOX2) couple EMT with cancer stem cell properties (Mitchell et al., 2023). Preclinical studies demonstrate that EZH2 inhibitors block this reprogramming, significantly improving paclitaxel response rates in PDX models (Yang et al., 2021). Non-coding RNAs amplify EMT heterogeneity through dual epigenetic-transcriptional control (Hashemi et al., 2022). For example, the long non-coding RNA MALAT1 recruits HDAC3 complexes to the CDH1 promoter, silencing its expression via histone deacetylation to promote invasion (Ferri et al., 2022). Clinically, epigenetic silencing of key EMT suppressors inversely correlates with enzalutamide resistance in CRPC patients, highlighting their potential as predictive biomarkers (Nikhil et al., 2020).

### EMT and drug resistance in PCa

Overcoming therapy resistance represents one of the most critical challenges in oncology, as it is a leading cause of treatment failure in tumors (Vasan et al., 2019). Therapy resistance encompasses rejection of chemotherapy, radiotherapy, immunotherapy, and targeted therapies, along with both inherent and acquired resistance to conventional tumor treatments (Vasan et al., 2019). The management of locally advanced prostate tumors relies on androgen deprivation therapy (ADT); however, clinical outcomes remain limited. Following the initial response, these patients inevitably progress to CRPC (Falagario et al., 2023; Preisser et al., 2024), which is connected with various forms of epithelial plasticity, including EMT (Dunning et al., 2011; Teng et al., 2021). To support this hypothesis, early studies have indicated that stem cells associated with castration resistance in PCa may arise from cells that hcluding tumor-associated macrophages, tumor-associated fibroblasts, regulatory T cells, and myeloid-derived suppressor cells (Sun et al., 2012). These immunosuppressive cell populations have also been shown to promote EMT in tumor cells through the secretion of inflammatory cytokines or exosomes (Sun et al., 2012). This interplay has been validated in various solid tumors, including PCa. In PCa, the expression of N-cadherin is significantly linked to immunoregulatory features. N-cadherin-positive regions exhibit a local decrease in intraepithelial cytotoxic (CD8) T cells and an increase in immunosuppressive regulatory T cells (Miao et al., 2017). This correlation is associated with the expression of the IDO1 protein and its metabolite, kynurenine, in the predominantly N-cadherin-positive regions. In summary, the dynamic interactions between EMT and the TME play a critical role in tumor progression and immune evasion. Understanding these interactions can offer valuable insights into potential therapeutic targets for enhancing cancer treatment outcomes (Sun et al., 2012). EMT markers, including N-cadherin, have been reported to be upregulated following androgen deprivation therapy and can directly induce the transition of PCa to CRPC (Miao et al., 2017; Mickova et al., 2021). Similarly, transcription factors related to EMT have also been implicated in CRPC. For instance, several studies have indicated that the EMT transcription factor Snail is related to with elevated Gleason scores, and its ectopic expression has been shown to result in increased levels of AR and AR variants. Additionally, Skp2-mediated stabilization of Twist not only facilitates the progression of PCa to CRPC but also correlates with resistance to paclitaxel in PCa (Mickova et al., 2021; Ruan et al., 2017). Furthermore, TGF-β, a major regulator of EMT, has been implicated in the development of CRPC. When its upstream inhibitor FOXA1 is lost, TGF-β expression and activity are upregulated, further facilitating the progression of CRPC (Wang et al., 2023). Inhibitors of TGF-β receptors have been shown to enhance the sensitivity of advanced PCa to enzalutamide treatment (Paller et al., 2019).

Similarly, EMT significantly impacts the sensitivity of solid tumors, including PCa, to chemotherapeutic agents, including docetaxel, paclitaxel, and epirubicin (Dunning et al., 2011; Teng et al., 2021). These agents are often utilized as first-line treatments for CRPC or its lethal subtypes following ADT failure (Falagario et al., 2023; Preisser et al., 2024). Although these agents demonstrate effective therapeutic outcomes initially, PCa inevitably develops resistance to them over time, similar to ADT. While the specific molecular mechanisms remain unclear, it is widely acknowledged that EMT influences the sensitivity of PCa to these agents. Laboratory evidence supports this assertion; previous studies have demonstrated that cells resistant to chemotherapeutic agents undergo epithelial-to-mesenchymal transition, resulting in decreased levels of E-cadherin and upregulation of mesenchymal markers (Du and Shim, 2016). Inhibiting the EMT process or reintroducing E-cadherin can restore sensitivity to chemotherapeutic agents (Ni et al., 2013; Hanrahan et al., 2017). Additionally, transcription factors related to EMT, including ZEB1 and SKP2, have been implicated in chemotherapy resistance by promoting EMT in PCa cells. In conclusion, overcoming therapy resistance, particularly by targeting EMT and its regulatory pathways, is essential for enhancing the efficacy of treatments for PCa and other solid tumors (Preisser et al., 2024; Dunning et al., 2011)^.^

### EMT and cancer stem cell (CSC) phenotype and spatial heterogeneity in PCa

Cell plasticity is regarded as a hallmark of EMT, wherein tumor epithelial cells undergoing EMT may exhibit intermediate morphological, transcriptional, and epigenetic characteristics. These features encompass a spectrum of both epithelial and mesenchymal markers, referred to as quasi-mesenchymal intermediates, and are believed to be primary contributors to EMT-associated cell plasticity, which has been linked to tumor stemness (Lambert and Weinberg, 2021). This notion is supported by findings that prostate tumor cells exhibiting mesenchymal characteristics demonstrate enhanced invasiveness and stemness (Soundararajan et al., 2018; Navas et al., 2020). CSC hypothesis is an emerging model that elucidates various molecular features of malignant tumors, along with their propensity for recurrence, metastasis, and resistance to conventional therapies. CSCs have been isolated from tumors in PCa patients and are considered one of the primary contributors to tumor resistance and recurrence (Batlle and Clevers, 2017). Observations indicate that the expression levels of tumor stemness markers tend to rise with tumor progression, and it has been shown that PCa neuroendocrine cells (AR and PSA negative) may be associated with CSCs (Verma et al., 2023). Notably, growing evidence suggests that the activation of the EMT process in PCa cells is linked to the acquisition and maintenance of stem cell properties within PCa epithelial cells (Soundararajan et al., 2018). N-cadherin has been demonstrated to elevate the expression of prostate cancer stem cell markers (c-Myc, Klf4, Sox2, and Oct4) through the ErbB signaling pathway, thereby enhancing the formation of prostate tumorospheres. The upregulation of cancer stem cell markers is linked to PCa cells displaying more pronounced mesenchymal traits. Similarly, in contrast to the CD44-negative population, mesenchymal markers, including N-cadherin and vimentin, are highly upregulated in the CD44-positive population. Prostate tumor cells exhibiting mesenchymal characteristics tend to demonstrate enhanced invasiveness and stemness (Wang et al., 2016). In summary, the interplay between EMT and CSCs underpins the plasticity of tumor cells, contributing to their aggressive behavior and resistance to therapies. Understanding these mechanisms is crucial for developing strategies to target these adaptive processes and enhance treatment outcomes for PCa (Fontana et al., 2019). Concurrently, endothelial cell-derived IL-8 activates the AKT/mTOR pathway via CXCR receptors, promoting the shift of hybrid epithelial-mesenchymal (E/M) cells toward mesenchymal phenotypes (Lu et al., 2025). Furthermore, glutamine deprivation in the tumor core activates GCN2 kinase, which phosphorylates eIF2α to suppress epithelial gene translation, driving mesenchymal transition (Lu et al., 2024). In EMT-active regions, M2 macrophages and regulatory T cells are significantly enriched compared to normal areas, fostering an immunosuppressive niche (Han et al., 2023). These cells secrete IL-10 and TGF-β to induce EMT in neighboring epithelial cells and shield mesenchymal-like cells from CD8^+^ T cell-mediated cytotoxicity (Denk et al., 2025). Hypoxic, TP53-mutated EMT clones in the tumor core rely on HIF-1α (Guan et al., 2021), while AR-V7-positive clones at the invasive front dominate via WNT signaling activation (Tsao et al., 2021). Such spatial heterogeneity in EMT dynamics, governed by multi-layered regulatory networks, may offer novel therapeutic strategies to overcome treatment resistance in Pca.

### EMT and the immunosuppressive microenvironment in PCa

The tumor microenvironment (TME) consists of various cell types, including cancer cells, infiltrating immune cells, and stromal cells, such as fibroblasts. Interactions among these different cell types can influence one another, thereby inducing changes in the TME that affect tumor progression and metastasis (Pitt et al., 2016; Aggarwal et al., 2021). Interestingly, there is an interaction between EMT and TME, two fundamental biological processes, and EMT is associated with the recruitment of immunosuppressive cell populations (tumor-associated macrophages, tumor-associated fibroblasts, regulatory T cells, and myeloid-derived suppressor cells) in TME (Aggarwal et al., 2021), and these immunosuppressive cell populations have also been reported to promote EMT in tumor cells by secreting cytokines or exosomes (Aggarwal et al., 2021), which has been demonstrated in a variety of solid tumors, including PCa, In PCa, N-cadherin expression is significantly associated with immunomodulatory profiles, with N-cadherin-positive regions exhibiting a local decrease in intraepithelial cytotoxic (CD8) T cells and an increase in immunosuppressive regulatory T cells (Sun et al., 2021), which is connected with the expression of IDO1 protein and its metabolite kynurenine in predominantly N-cadherin-positive regions (Sun et al., 2021).

## Clinical correlation between EMT and prostate cancer

Recent advances in targeting EMT transcription factors have yielded promising clinical validation of Twist1 and Snail inhibitors in metastatic castration-resistant prostate cancer (mCRPC) (Taki et al., 2021). Studies demonstrate that combining Harmine derivatives with enzalutamide reduces Twist1 mRNA levels in mCRPC patients, correlating with prolonged radiographic progression-free survival (rPFS) (Dellal et al., 2020; Agarwal et al., 2023). However, while the STAT3/Snail dual-pathway inhibitor Napabucasin upregulates E-cadherin in treated tissues (Li et al., 2024), its use is limited by increased diarrhea incidence (Wang S. T. et al., 2024). The histone deacetylase inhibitor Vorinostat, combined with abiraterone, significantly extends overall survival in mCRPC patients with high EMT scores (Tanabe et al., 2024). This heterogeneity aligns with genome-wide methylation analyses, where ZEB1 and VIM demethylation strongly associates with PSA decline (Rajamäki et al., 2021; Davies et al., 2023). Interim analyses of TGF-β inhibitor/PD-1 antibody combinations in high-EMT mCRPC reveal rising objective response rates, with reduced EMT scores linked to enhanced intratumoral CD8^+^ T cell infiltration (Lin et al., 2021).

## Pathways associated with EMT activation in PCa cells

### Transforming growth factor beta (TGF-β)

TGF-β is a multifunctional cytokine synthesized by various tissue cells (Massagué and Sheppard, 2023). The TGF-β signaling pathway encompasses various multifunctional cytokines, their corresponding receptors, and intracellular signal transduction molecules, and is associated with several cellular activities, including EMT, growth arrest, and tissue fibrosis (Massagué and Sheppard, 2023). It has been established as a primary regulatory factor for EMT activation in PCa (Mirzaei et al., 2022). TGF-β promotes EMT activation in PCa by inducing vimentin and fibronectin while inhibiting E-cadherin levels, which is associated with the upregulation of EMT transcription factors following activation of the classical TGF-β signaling pathway (Smad-dependent pathway) (Mirzaei et al., 2022). The expression level of this protein is typically correlated with poorer prognosis and higher expression levels in PCa specimens (Torrealba et al., 2020). Conversely, negative regulators of TGF-β have been reported to exhibit decreased expression levels with the progression of PCa. For instance, FOXA1 (a negative regulator of neuroendocrine differentiation) displays lower expression levels in CRPC patients, which is linked to active TGF-β-induced cellular events, including EMT, suggesting potential activation of the EMT process in CRPC (Song et al., 2019). Similarly, another critical regulator of TGF-β effects is SOX5, which initiates EMT by binding to the TWIST1 promoter. A reduction in the expression level of this molecule results in the loss of the mesenchymal phenotype and migratory capacity of PCa cells (Hu et al., 2018). Furthermore, TGF-β has been shown to promote EMT activation in PCa through additional oncogenic pathways. For instance, TGF-β can initiate the EMT process in PCa by activating the PI3K/Akt signaling pathway, which dissociates the E-cadherin/catenin complex from the actin cytoskeleton (Torrealba et al., 2020; Song et al., 2019; Hu et al., 2018). In summary, TGF-β plays a crucial role in EMT activation in PCa through various pathways, including the Smad-dependent pathway and other oncogenic signaling pathways such as PI3K/Akt (Mirzaei et al., 2022; Torrealba et al., 2020; Song et al., 2019; Hu et al., 2018; Hamidi et al., 2017). Understanding these mechanisms is essential for developing therapeutic strategies aimed at targeting EMT and enhancing treatment outcomes for PCa.

### AR signaling pathway and ADT therapy

PCa is primarily an androgen-dependent disease. The standard therapy for metastatic PCa involves inhibiting androgen synthesis, lowering circulating androgen levels, and blocking the AR (Falagario et al., 2023; Preisser et al., 2024). Previous literature has reported several clinical trial outcomes for second-generation ADT drugs. These drugs target the AR ligand-binding domain and, although they initially achieve significant therapeutic effects, they inevitably lead to resistance over time (Preisser et al., 2024; Dunning et al., 2011). This resistance is linked to changes in AR expression, the emergence of variant receptors, and specific mutations (Zheng et al., 2022). Notably, the relationship between the AR signaling pathway and EMT is intricate and not fully understood. Previous studies have indicated that AR can induce the expression of various proteases, including MMP2/9, and promote cellular invasion, which is linked to EMT activation (Li et al., 2007). Consequently, early research suggested that ADT might inhibit EMT in PCa. However, practical studies reveal a more complex relationship. ADT has been shown to induce EMT genes, with PCa cells exhibiting upregulated EMT-related genes and mesenchymal phenotypes following ADT treatment (Chen et al., 2017). AR-negative PCa cell lines (PC3 and DU145) demonstrate higher mesenchymal gene expression and reduced epithelial characteristics compared to androgen-dependent PCa cell lines (LNCaP) (Moll et al., 2022). This phenomenon may be attributed to ADT treatment diminishing AR’s inhibition of Sail genes, thereby promoting PCa EMT (Moll et al., 2022; Cmero et al., 2021). However, some studies indicate that EMT induction during ADT treatment may result from the upregulation of the chemokine CCL2 (Lee et al., 2018). CCL2 is known to enhance cell motility and is recognized as a paracrine regulator that promotes tumor metastasis (sai et al., 2018). Its expression is elevated during ADT treatment. Moreover, the emergence of AR variants following ADT treatment can influence EMT gene expression. For instance, AR-V7 has been demonstrated to induce mesenchymal genes and tumor stemness, fostering metastasis (Lee et al., 2018). Furthermore, emerging research highlights that estrogen receptor (ER) signaling drives disease progression following AR deprivation, with compensatory ER pathway activation playing a critical role. Specifically, the ERα subtype promotes tumor proliferation and bone metastasis via MAPK/ERK activation, correlating with reduced overall survival (Erdmann et al., 2022). In contrast, ERβ suppresses tumorigenesis by inhibiting NF-κB signaling (Yang J. et al., 2020). AR inhibition disrupts the ERα/ERβ balance, reshaping tumor biology (Wang et al., 2021). Post-AR suppression, ERα upregulation drives proliferation and induces EMT through MAPK/ERK activation (Chakraborty et al., 2023). While ERβ overexpression inhibits localized PCa growth in animal models, this protective effect diminishes after AR inhibition (Wang et al., 2022). Compensatory ER signaling triggered by AR-targeted therapies remodels tumor evolution and microenvironment adaptation via ERα/ERβ imbalance (Liu Q. et al., 2023). In summary, androgen deprivation therapy intricately links ER signaling to EMT in PCa. However, modulating AR or ER to control EMT remains a key challenge in advancing therapeutic strategies.

### Inflammation and cytokines

EMT can also be induced by specific inflammatory factors and cytokines (Wang P. et al., 2024). IL-6, a cytokine belonging to the chemokine family, has been found at elevated levels in metastatic specimens from PCa patients and is associated with a mesenchymal phenotype in PCa (Wang P. et al., 2024; Nguyen et al., 2014). This indicates that IL-6 may act as an effective inducer of EMT in PCa. Moreover, TNF-α, a dimeric soluble cytokine, is regarded as a regulatory factor in EMT associated with PCa. Activation of the AKT/GSK-3β signaling pathway in PCa stabilizes Snail, a key mechanism driving EMT in tumor cells (Fontanella et al., 2021). Notably, the fibroblast growth factor (FGF) family has been linked to EMT in PCa. An *in vitro* study using PCa cell lines demonstrated that FGF2 enhances mesenchymal markers while reducing epithelial markers, thereby inducing EMT and promoting increased cellular invasion and metastasis (Wang et al., 2013). Mechanistically, this may result from FGF9 activating c-Jun-dependent TGF-β secretion in prostate stromal cells, which subsequently triggers EMT in prostate cancer cells through paracrine signaling (Wang et al., 2013; Huang et al., 2015; Jin et al., 2004). In summary, several cytokines and growth factors, including IL-6, TNF-α, and members of the FGF family, play crucial roles in the activation of EMT in PCa. Understanding the specific pathways and mechanisms by which these factors influence EMT could provide valuable insights for developing targeted therapies aimed at mitigating cancer progression and metastasis.

### Treatment strategies related to EMT

Therapeutic strategies targeting EMT primarily focus on biomarkers capable of predicting disease outcomes. Recent studies have employed metrics such as hazard ratios (HRs) and receiver operating characteristic (ROC) curves to quantify the clinical predictive value of EMT biomarkers, though findings vary significantly across studies (Mazumder et al., 2022). Retrospective analyses using Cox proportional hazards models confirm that expression levels of core EMT transcription factors (Twist, Snail) and adhesion molecules (E-cadherin, N-cadherin) correlate strongly with patient survival outcomes (Qu et al., 2021). For instance, Twist overexpression associates with reduced progression-free survival (Shen et al., 2022), while Snail positivity increases mortality risk in colorectal cancer (Bao et al., 2022). Notably, prognostic implications differ by cancer type: Vimentin predicts poor outcomes in lung cancer (Bronte et al., 2021) but exhibits protective effects in PCa (Choudhry et al., 2023). ROC analyses further demonstrate that biomarker combinations like the E-cadherin/Vimentin ratio show promising sensitivity and specificity for early metastasis detection (Lee et al., 2020).

In PCa, epithelial plasticity is directly linked to poor clinical prognosis (Lee et al., 2020). Genomic analyses have identified distinct genetic signatures linked to various intermediate states of EMT, aiding in the identification of EMT-related transcription factors that can serve as biomarkers. For instance, a study by Jedroszka and colleagues classified patients into several groups based on their expression levels of AR, ESR1, and ESR2 (Zhang et al., 2014; Jędroszka et al., 2017). After analyzing 43 genes involved in EMT, they found that variations in gene expression could predict more aggressive phenotypes in individuals under 50 years old (Jędroszka et al., 2017). Similarly, CTCs exhibiting an EMT phenotype can predict disease recurrence and metastasis (Liu et al., 2020). Furthermore, prior studies have reported a risk scoring model derived from EMT-related genes to predict disease outcomes, demonstrating significant clinical benefits (Feng et al., 2022). Similar risk scoring models have also been reported in other tumors (He et al., 2023; Yang C. et al., 2020; Zhang Z. J. et al., 2023). These findings suggest that future research may specifically target patients with high expression of mesenchymal markers, exploring personalized immunotherapy for molecular subtypes exhibiting varying levels of EMT gene expression. As previously mentioned, in PCa, EMT appears to be a key mediator of acquired resistance to androgen deprivation and docetaxel therapies. EMT activation following ADT treatment is linked to castration resistance. Targeting or reversing the EMT process in epithelial cancer cells undergoing ADT treatment could be a promising strategy for enhancing PCa outcomes. Metformin, a widely used antidiabetic medication, has demonstrated effective antitumor activity (Yu and Suissa, 2023). Prior studies have indicated that it can reverse resistance to enzalutamide by targeting two key pathways closely associated with EMT regulation (TGF-β and STAT3), highlighting the feasibility of combining EMT inhibitors with ADT therapy (Liu et al., 2017). This is supported by several additional studies, including those demonstrating that cabazitaxel and TGF-β receptor inhibitors enhance sensitivity to enzalutamide and reverse the EMT process when combined with ADT treatment (Paller et al., 2019; Song et al., 2019). EMT activation impairs DNA repair capacity, increasing tumor mutational burden (Moyret-Lalle et al., 2022). In PCa metastases, EMT-high cells harbor more nonsynonymous mutations than epithelial counterparts, with enrichment in PI3K/AKT and WNT pathways driving clonal evolution (Therachiyil et al., 2022). Abiraterone treatment significantly expands EMT-driven AR-V7 splice variant-positive clones, revealing co-evolution between EMT and castration resistance (Gurioli et al., 2022). EMT reversibility enables phenotypic switching under therapeutic pressure (Shu et al., 2020). Animal models show transient increases in Vimentin+ cells within docetaxel-treated PCa bone metastases, which revert to baseline post-treatment, correlating with drug concentration gradients (Wei et al., 2024). Notably, EMT plasticity activates SOX2/OCT4 pathways to confer cancer stem cell-like properties, enhancing residual cell regeneration and drug tolerance (Marques-Magalhães et al., 2025). Clinically, patients with fluctuating EMT scores exhibit elevated recurrence risk (Kim et al., 2025). By remodeling the extracellular matrix (ECM) and fostering immunosuppressive microenvironments, EMT creates niches that favor resistant clone expansion (Wang Y. et al., 2024). Moreover, the selective estrogen receptor modulator raloxifene has been identified as a potential treatment for PCa. *In vivo* studies have demonstrated that this drug inhibits EMT by suppressing N-cadherin, SLUG, SNAIL, vimentin, and matrix metalloproteinases, thereby impacting the efficacy of ADT treatment (Di Zazzo et al., 2019).

Recent breakthroughs in detecting circulating tumor cells (CTCs) exhibiting EMT features have significantly improved prognostic accuracy in PCa. These advancements leverage multimodal capture strategies and molecular profiling to enhance detection reliability (Cai et al., 2024). Traditional CTC detection relies on epithelial markers like EpCAM. However, EMT-induced loss of these markers often leads to undetected mesenchymal CTCs, limiting diagnostic sensitivity (Di Zazzo et al., 2019). Emerging technologies address this limitation by integrating physical properties, functional biomarkers, and single-cell analysis, enabling precise capture and molecular characterization of EMT-heterogeneous CTCs (Guan et al., 2024). For example, the “Tri-Modal Chip” combines EpCAM antibody coatings, size-based filtration, and deformability analysis. This method improves CTC detection rates in mCRPC and distinguishes epithelial, hybrid, and mesenchymal CTC subtypes (Li et al., 2025). Studies indicate that patients with higher proportions of mesenchymal CTCs (M-CTCs) experience shorter progression-free survival (PFS) (Du et al., 2024). The aggressive behavior of mesenchymal CTCs correlates with their metabolic activity. New platforms now evaluate metabolic states using JC-1 mitochondrial membrane potential staining and lactate secretion measurements (Guder et al., 2024). Notably, CTCs with elevated lactate secretion strongly associate with bone metastasis risk in PCa, outperforming PSA levels as a predictive biomarker (Bergmann et al., 2025). Furthermore, real-time monitoring of CTC glycolytic activity via extracellular acidification rate (ECAR) analysis provides functional evidence for dynamic EMT progression (Luan et al., 2024).

In summary, these findings indicate that targeting the EMT process in PCa may represent an effective therapeutic strategy. However, a comprehensive understanding of the molecular biology of EMT and its relationship with established molecular subtypes of advanced PCa is essential for effectively implementing EMT-targeted therapeutic strategies in clinical trials.

## Conclusion and future directions

The era of personalized precision medicine has begun in oncology, yet enhancing patient survival and improving prognosis continue to pose significant challenges. Research on EMT highlights the importance of understanding the interplay between cellular plasticity, stemness, and treatment response. These processes are closely associated with tumorigenesis, invasiveness, migration, metastasis, and interactions with TME. EMT can be induced by various drivers and effectors, which may serve as prognostic biomarkers or targets for interventions in metastatic disease. Furthermore, the role of EMT in cancer progression and therapeutic resistance has been extensively investigated. In PCa, EMT is upregulated after ADT and is correlated with the emergence of CRPC. This implies that future translational research on the “reprogramming” of cancer cell fate through EMT could empower lethal PCa to overcome treatment resistance and improve patient survival. Unfortunately, there are currently few therapeutic agents or methods capable of effectively modulating the EMT process to enhance the efficacy of cancer treatments. In cellular and animal studies, targeting specific transcriptional genes has been shown to reverse EMT and enhance tumor treatment sensitivity. However, achieving long-term success in humans remains fraught with considerable risks and challenges.

## References

[B1] Agarwal N. Azad A. A. Carles J. Fay A. P. Matsubara N. Heinrich D. (2023). Talazoparib plus enzalutamide in men with first-line metastatic castration-resistant prostate cancer (TALAPRO-2): a randomised, placebo-controlled, phase 3 trial. Lancet 402 (10398), 291–303. 10.1016/S0140-6736(23)01055-3 37285865

[B2] Aggarwal V. Montoya C. A. Donnenberg V. S. Sant S. (2021). Interplay between tumor microenvironment and partial EMT as the driver of tumor progression. iScience 24 (2), 102113. 10.1016/j.isci.2021.102113 33659878 PMC7892926

[B3] Akhmetkaliyev A. Alibrahim N. Shafiee D. Tulchinsky E. (2023). EMT/MET plasticity in cancer and Go-orGrow decisions in quiescence: the two sides of the same coin? Mol. Cancer 22 (1), 90. 10.1186/s12943-023-01793-z 37259089 PMC10230810

[B4] Bao Z. Zeng W. Zhang D. Wang L. Deng X. Lai J. (2022). SNAIL induces EMT and lung metastasis of tumours secreting CXCL2 to promote the invasion of M2-type immunosuppressed macrophages in colorectal cancer. Int. J. Biol. Sci. 18 (7), 2867–2881. 10.7150/ijbs.66854 35541899 PMC9066124

[B5] Batlle E. Clevers H. (2017). Cancer stem cells revisited. Nat. Med. 23 (10), 1124–1134. 10.1038/nm.4409 28985214

[B6] Baumeister P. Zhou J. Canis M. Gires O. (2021). Epithelial-to-Mesenchymal transition-derived heterogeneity in head and neck squamous cell carcinomas. Cancers (Basel) 13 (21), 5355. 10.3390/cancers13215355 34771518 PMC8582421

[B7] Bergmann L. Greimeier S. Riethdorf S. Rohlfing T. Kaune M. Busenbender T. (2025). Transcriptional profiles of circulating tumor cells reflect heterogeneity and treatment resistance in advanced prostate cancer. J. Exp. Clin. Cancer Res. 44 (1), 111. 10.1186/s13046-025-03367-x 40181402 PMC11967125

[B8] Bhat S. Kabekkodu S. P. Adiga D. Fernandes R. Shukla V. Bhandari P. (2021). ZNF471 modulates EMT and functions as methylation regulated tumor suppressor with diagnostic and prognostic significance in cervical cancer. Cell Biol. Toxicol. 37 (5), 731–749. 10.1007/s10565-021-09582-4 33566221 PMC8490246

[B9] Bronte G. Puccetti M. Petracci E. Landi L. Cravero P. Scodes S. (2021). The interplay between programmed death ligand 1 and vimentin in advanced non-small-cell lung cancer. Front. Oncol. 11, 669839. 10.3389/fonc.2021.669839 34017688 PMC8130554

[B10] Cai J. Zhang W. Lu Y. Liu W. Zhou H. Liu M. (2024). Single-cell exome sequencing reveals polyclonal seeding and TRPS1 mutations in colon cancer metastasis. Signal Transduct. Target Ther. 9 (1), 247. 10.1038/s41392-024-01960-8 39307879 PMC11417107

[B11] Carroll J. D. Mack M. J. Vemulapalli S. Herrmann H. C. Gleason T. G. Hanzel G. (2020). STS-ACC TVT registry of transcatheter aortic valve replacement. J. Am. Coll. Cardiol. 76 (21), 2492–2516. 10.1016/j.jacc.2020.09.595 33213729

[B12] Cha H. R. Lee J. H. Ponnazhagan S. (2020). Revisiting immunotherapy: a focus on prostate cancer. Cancer Res. 80 (8), 1615–1623. 10.1158/0008-5472.CAN-19-2948 32066566 PMC7641094

[B13] Chakraborty B. Byemerwa J. Krebs T. Lim F. Chang C. Y. McDonnell D. P. (2023). Estrogen receptor signaling in the immune system. Endocr. Rev. 44 (1), 117–141. 10.1210/endrev/bnac017 35709009

[B14] Chen J. R. Caviness P. C. Zhao H. Belcher B. Wankhade U. D. Shankar K. (2022). Maternal high-fat diet modifies epigenetic marks H3K27me3 and H3K27ac in bone to regulate offspring osteoblastogenesis in mice. Epigenetics 17 (13), 2209–2222. 10.1080/15592294.2022.2111759 35950595 PMC9665156

[B15] Chen W. Y. Tsai Y. C. Yeh H. L. Suau F. Jiang K. C. Shao A. N. (2017). Loss of SPDEF and gain of TGFBI activity after androgen deprivation therapy promote EMT and bone metastasis of prostate cancer. Sci. Signal 10 (492), eaam6826. 10.1126/scisignal.aam6826 28811384

[B16] Choudhry M. Gamallat Y. Khosh Kish E. Seyedi S. Gotto G. Ghosh S. (2023). *Downregulation of BUD31* promotes prostate cancer cell proliferation and migration via activation of p-AKT and vimentin *in vitro* . Int. J. Mol. Sci. 24 (7), 6055. 10.3390/ijms24076055 37047027 PMC10094631

[B17] Cmero M. Kurganovs N. J. Stuchbery R. McCoy P. Grima C. Ngyuen A. (2021). Loss of SNAI2 in prostate cancer correlates with clinical response to androgen deprivation therapy. JCO Precis. Oncol. 5, 1048–1059. 10.1200/PO.20.00337 PMC8238292 34322653

[B18] Davies C. R. Guo T. Burke E. Stankiewicz E. Xu L. Mao X. (2023). The potential of using circulating tumour cells and their gene expression to predict docetaxel response in metastatic prostate cancer. Front. Oncol. 12, 1060864. 10.3389/fonc.2022.1060864 36727071 PMC9885040

[B19] Dellal H. Boulahtouf A. Alaterre E. Cuenant A. Grimaldi M. Bourguet W. (2020). High content screening using new U2OS reporter cell models identifies harmol hydrochloride as a selective and competitive antagonist of the androgen receptor. Cells 9 (6), 1469. 10.3390/cells9061469 32560058 PMC7349874

[B20] Denk D. Ramakrishnan M. Conche C. Pallangyo C. Pesic M. Ceteci F. (2025). IL-17RA signaling provides dual tumor-suppressor function during late-stage colorectal carcinogenesis. Immunity 58 (3), 701–715.e8. 10.1016/j.immuni.2025.02.005 40023157

[B21] Di Zazzo E. Galasso G. Giovannelli P. Di Donato M. Bilancio A. Perillo B. (2019). Estrogen receptors in epithelial-mesenchymal transition of prostate cancer. Cancers (Basel) 11 (10), 1418. 10.3390/cancers11101418 31548498 PMC6826537

[B22] Du B. Shim J. S. (2016). Targeting epithelial-mesenchymal transition (EMT) to overcome drug resistance in cancer. Molecules 21 (7), 965. 10.3390/molecules21070965 27455225 PMC6273543

[B23] Du D. Liu C. Qin M. Zhang X. Xi T. Yuan S. (2022). Metabolic dysregulation and emerging therapeutical targets for hepatocellular carcinoma. Acta Pharm. Sin. B 12 (2), 558–580. 10.1016/j.apsb.2021.09.019 35256934 PMC8897153

[B24] Du K. Y. Wu S. Ma X. Liu Y. (2024). Circulating tumor cell phenotype detection based on epithelial-mesenchymal transition markers combined with clinicopathological risk has potential to better predict recurrence in stage III breast cancer treated with neoadjuvant chemotherapy: a pilot study. Breast Cancer Res. Treat. 207 (3), 517–527. 10.1007/s10549-024-07430-7 38990453

[B25] Du X. Hu H. (2021). The roles of 2-hydroxyglutarate. Front. Cell Dev. Biol. 9, 651317. 10.3389/fcell.2021.651317 33842477 PMC8033037

[B26] Dunning N. L. Laversin S. A. Miles A. K. Rees R. C. (2011). Immunotherapy of prostate cancer: should we be targeting stem cells and EMT? Cancer Immunol. Immunother. 60 (8), 1181–1193. 10.1007/s00262-011-1065-8 21688178 PMC11029142

[B27] Erdmann É. Ould Madi Berthélémy P. Cottard F. Angel C. Z. Schreyer E. Ye T. (2022). Androgen receptor-mediated transcriptional repression targets cell plasticity in prostate cancer. Mol. Oncol. 16 (13), 2518–2536. 10.1002/1878-0261.13164 34919781 PMC9462842

[B28] Falagario U. G. Abbadi A. Remmers S. Björnebo L. Bogdanovic D. Martini A. (2023). Biochemical recurrence and risk of mortality following radiotherapy or radical prostatectomy. JAMA Netw. Open 6 (9), e2332900. 10.1001/jamanetworkopen.2023.32900 37695584 PMC10495864

[B29] Feng D. Shi X. Xiong Q. Zhang F. Li D. Yang L. (2022). A gene prognostic index associated with EpithelialMesenchymal transition predicting biochemical recurrence and tumor chemoresistance for prostate cancer. Front. Oncol. 11, 805571. 10.3389/fonc.2021.805571 35096608 PMC8790245

[B30] Ferri C. Di Biase A. Bocchetti M. Zappavigna S. Wagner S. Le Vu P. (2022). MiR-423-5p prevents MALAT1-mediated proliferation and metastasis in prostate cancer. J. Exp. Clin. Cancer Res. 41 (1), 20. 10.1186/s13046-021-02233-w 35016717 PMC8751098

[B31] Fontana F. Anselmi M. Limonta P. (2023). Unraveling the peculiar features of mitochondrial metabolism and dynamics in prostate cancer. Cancers (Basel) 15 (4), 1192. 10.3390/cancers15041192 36831534 PMC9953833

[B32] Fontana F. Raimondi M. Marzagalli M. Sommariva M. Limonta P. Gagliano N. (2019). Epithelial-to-mesenchymal transition markers and CD44 isoforms are differently expressed in 2D and 3D cell cultures of prostate cancer cells. Cells 8 (2), 143. 10.3390/cells8020143 30754655 PMC6406374

[B33] Fontanella R. A. Sideri S. Di Stefano C. Catizone A. Di Agostino S. Angelini D. F. (2021). CD44v8-10 is a marker for malignant traits and a potential driver of bone metastasis in a subpopulation of prostate cancer cells. Cancer Biol. Med. 18 (3), 788–807. 10.20892/j.issn.2095-3941.2020.0495 34018387 PMC8330537

[B34] Gillessen S. Bossi A. Davis I. D. de Bono J. Fizazi K. James N. D. (2023). Management of patients with advanced prostate cancer. Part I: intermediate-/high-risk and locally advanced disease, biochemical relapse, and side effects of hormonal treatment: report of the advanced prostate cancer consensus conference 2022. Eur. Urol. 83 (3), 267–293. 10.1016/j.eururo.2022.11.002 36494221 PMC7614721

[B35] Guan D. Li C. Li Y. Li Y. Wang G. Gao F. (2021). The DpdtbA induced EMT inhibition in gastric cancer cell lines was through ferritinophagy-mediated activation of p53 and PHD2/hif-1α pathway. J. Inorg. Biochem. 218, 111413. 10.1016/j.jinorgbio.2021.111413 33713969

[B36] Guan Y. Liu X. Tian J. Yang G. Xu F. Guo N. (2024). CCL5 promotes the epithelial-mesenchymal transition of circulating tumor cells in renal cancer. J. Transl. Med. 22 (1), 817. 10.1186/s12967-024-05297-2 39227943 PMC11370314

[B37] Guder C. Heinrich S. Seifert-Klauss V. Kiechle M. Bauer L. Öllinger R. (2024). Extracellular Hsp70 and circulating endometriotic cells as novel biomarkers for endometriosis. Int. J. Mol. Sci. 25 (21), 11643. 10.3390/ijms252111643 39519195 PMC11546379

[B38] Gurioli G. Conteduca V. Brighi N. Scarpi E. Basso U. Fornarini G. (2022). Circulating tumor cell gene expression and plasma AR gene copy number as biomarkers for castration-resistant prostate cancer patients treated with cabazitaxel. BMC Med. 20 (1), 48. 10.1186/s12916-022-02244-0 35101049 PMC8805338

[B39] Haffner M. C. Zwart W. Roudier M. P. True L. D. Nelson W. G. Epstein J. I. (2021). Genomic and phenotypic heterogeneity in prostate cancer. Nat. Rev. Urol. 18 (2), 79–92. 10.1038/s41585-020-00400-w 33328650 PMC7969494

[B40] Hamidi A. Song J. Thakur N. Itoh S. Marcusson A. Bergh A. (2017). TGF-β promotes PI3K-AKT signaling and prostate cancer cell migration through the TRAF6-mediated ubiquitylation of p85α. Sci. Signal 10 (486), eaal4186. 10.1126/scisignal.aal4186 28676490

[B41] Han S. Bao X. Zou Y. Wang L. Li Y. Yang L. (2023). d-lactate modulates M2 tumor-associated macrophages and remodels immunosuppressive tumor microenvironment for hepatocellular carcinoma. Sci. Adv. 9 (29), eadg2697. 10.1126/sciadv.adg2697 37467325 PMC10355835

[B42] Hanrahan K. O'Neill A. Prencipe M. Bugler J. Murphy L. Fabre A. (2017). The role of epithelial-mesenchymal transition drivers ZEB1 and ZEB2 in mediating docetaxel-resistant prostate cancer. Mol. Oncol. 11 (3), 251–265. 10.1002/1878-0261.12030 28133913 PMC5527446

[B43] Hashemi M. Moosavi M. S. Abed H. M. Dehghani M. Aalipour M. Heydari E. A. (2022). Long non-coding RNA (lncRNA) H19 in human cancer: from proliferation and metastasis to therapy. Pharmacol. Res. 184, 106418. 10.1016/j.phrs.2022.106418 36038043

[B44] He R. Lu J. Feng J. Lu Z. Shen K. Xu K. (2024). Advancing immunotherapy for melanoma: the critical role of single-cell analysis in identifying predictive biomarkers. Front. Immunol. 15, 1435187. 10.3389/fimmu.2024.1435187 39026661 PMC11254669

[B45] He S. Li X. Zhou X. Weng W. Lai J. (2023). Role of epithelial cell-mesenchymal transition regulators in molecular typing and prognosis of colon cancer. J. Gastrointest. Oncol. 14 (2), 744–757. 10.21037/jgo-23-49 37201067 PMC10186519

[B46] Hu J. Tian J. Zhu S. Sun L. Yu J. Tian H. (2018). Sox5 contributes to prostate cancer metastasis and is a master regulator of TGF-β-induced epithelial mesenchymal transition through controlling Twist1 expression. Br. J. Cancer 118 (1), 88–97. 10.1038/bjc.2017.372 29123266 PMC5765224

[B47] Huang Y. Jin C. Hamana T. Liu J. Wang C. An L. (2015). Overexpression of FGF9 in prostate epithelial cells augments reactive stroma formation and promotes prostate cancer progression. Int. J. Biol. Sci. 11 (8), 948–960. 10.7150/ijbs.12468 26157349 PMC4495412

[B48] Icard P. Simula L. Fournel L. Leroy K. Lupo A. Damotte D. (2022). The strategic roles of four enzymes in the interconnection between metabolism and oncogene activation in non-small cell lung cancer: therapeutic implications. Drug Resist Updat 63, 100852. 10.1016/j.drup.2022.100852 35849943

[B49] Jędroszka D. Orzechowska M. Hamouz R. Górniak K. Bednarek A. K. (2017). Markers of epithelial-to-mesenchymal transition reflect tumor biology according to patient age and Gleason score in prostate cancer. PLoS One 12 (12), e0188842. 10.1371/journal.pone.0188842 29206234 PMC5714348

[B50] Jia H. Chen X. Zhang L. Chen M. (2025). Cancer associated fibroblasts in cancer development and therapy. J. Hematol. Oncol. 18 (1), 36. 10.1186/s13045-025-01688-0 40156055 PMC11954198

[B51] Jin C. Wang F. Wu X. Yu C. Luo Y. McKeehan W. L. (2004). Directionally specific paracrine communication mediated by epithelial FGF9 to stromal FGFR3 in two-compartment premalignant prostate tumors. Cancer Res. 64 (13), 4555–4562. 10.1158/0008-5472.CAN-03-3752 15231666

[B52] Kao K. C. Vilbois S. Tsai C. H. Ho P. C. (2022). Metabolic communication in the tumour-immune microenvironment. Nat. Cell Biol. 24 (11), 1574–1583. 10.1038/s41556-022-01002-x 36229606

[B53] Kim H. S. Yoon J. H. Choi J. Y. Yoon M. G. Baek G. O. Kang M. (2025). GULP1 as a novel diagnostic and predictive biomarker in hepatocellular carcinoma. Clin. Mol. Hepatol. 10.3350/cmh.2024.1038 PMC12260647 39914372

[B54] Lambert A. W. Weinberg R. A. (2021). Linking EMT programmes to normal and neoplastic epithelial stem cells. Nat. Rev. Cancer 21 (5), 325–338. 10.1038/s41568-021-00332-6 33547455

[B55] Lee G. T. Kwon S. J. Kim J. Kwon Y. S. Lee N. Hong J. H. (2018). WNT5A induces castration-resistant prostate cancer via CCL2 and tumour-infiltrating macrophages. Br. J. Cancer 118 (5), 670–678. 10.1038/bjc.2017.451 29381686 PMC5846063

[B56] Lee J. You J. H. Kim M. S. Roh J. L. (2020). Epigenetic reprogramming of epithelial-mesenchymal transition promotes ferroptosis of head and neck cancer. Redox Biol. 37, 101697. 10.1016/j.redox.2020.101697 32896720 PMC7484553

[B57] Li B. Y. Liao X. B. Fujito A. Thrasher J. B. Shen F. Y. Xu P. Y. (2007). Dual androgen-response elements mediate androgen regulation of MMP-2 expression in prostate cancer cells. Asian J. Androl. 9 (1), 41–50. 10.1111/J.1745-7262.2007.00226.x 16888681

[B58] Li H. Li J. Zhang Z. Yang Q. Du H. Dong Q. (2025). Digital quantitative detection for heterogeneous protein and mRNA expression patterns in circulating tumor cells. Adv. Sci. (Weinh) 12 (2), e2410120. 10.1002/advs.202410120 39556692 PMC11727120

[B59] Li Y. Zhong Y. Li C. Han Z. Cui Y. He R. (2024). Interleukin-9 promotes EMT-mediated PM_2.5_-induced pulmonary fibrosis by activating the STAT3 pathway. Arch. Toxicol. 98 (12), 4047–4058. 10.1007/s00204-024-03864-6 39259283

[B60] Lin B. B. Lei H. Q. Xiong H. Y. Fu X. Shi F. Yang X. W. (2021). MicroRNA-regulated transcriptome analysis identifies four major subtypes with prognostic and therapeutic implications in prostate cancer. Comput. Struct. Biotechnol. J. 19, 4941–4953. 10.1016/j.csbj.2021.08.046 34527198 PMC8433071

[B61] Liu H. Ding J. Wu Y. Wu D. Qi J. (2020). Prospective study of the clinical impact of epithelial and mesenchymal circulating tumor cells in localized prostate cancer. Cancer Manag. Res. 12, 4549–4560. 10.2147/CMAR.S253997 32606948 PMC7304675

[B62] Liu Q. Liu Y. Li X. Wang D. Zhang A. Pang J. (2023). Perfluoroalkyl substances promote breast cancer progression via ERα and GPER mediated PI3K/Akt and MAPK/Erk signaling pathways. Ecotoxicol. Environ. Saf. 258, 114980. 10.1016/j.ecoenv.2023.114980 37148752

[B63] Liu Q. Tong D. Liu G. Xu J. Do K. Geary K. (2017). Metformin reverses prostate cancer resistance to enzalutamide by targeting TGF-β1/STAT3 axis-regulated EMT. Cell Death Dis. 8 (8), e3007. 10.1038/cddis.2017.417 28837141 PMC5596596

[B64] Liu Y. Yu K. Kong X. Zhang K. Wang L. Zhang N. (2023). FOXA1 O-GlcNAcylation-mediated transcriptional switch governs metastasis capacity in breast cancer. Sci. Adv. 9 (33), eadg7112. 10.1126/sciadv.adg7112 37595040 PMC10438466

[B65] Lu C. Zhang Y. Sun C. Na Y. Sun H. Ma J. (2025). Stromal cell derived factor-1 promotes hepatic insulin resistance via inhibiting hepatocyte lipophagy. J. Cell Mol. Med. 29 (2), e70352. 10.1111/jcmm.70352 39855896 PMC11761003

[B66] Lu H. J. Koju N. Sheng R. (2024). Mammalian integrated stress responses in stressed organelles and their functions. Acta Pharmacol. Sin. 45 (6), 1095–1114. 10.1038/s41401-023-01225-0 38267546 PMC11130345

[B67] Lu L. Hu W. Liu B. Yang T. (2022). Insights into circulating tumor cell clusters: a barometer for treatment effects and prognosis for prostate cancer patients. Cancers (Basel) 14 (16), 3985. 10.3390/cancers14163985 36010983 PMC9406494

[B68] Luan H. Chen S. Lian J. Zhao B. Xu X. Chen Y. (2024). Biofluorescence imaging-guided spatial metabolic tracing: *in vivo* tracking of metabolic activity in circulating tumor cell-mediated multi-organ metastases. Talanta 280, 126696. 10.1016/j.talanta.2024.126696 39137660

[B69] Marques-Magalhães Â. Monteiro-Ferreira S. Canão P. A. Rios E. Costa Â. M. Castro F. (2025). Patient-derived colorectal cancer extracellular matrices modulate cancer cell stemness markers. Int. J. Mol. Sci. 26 (7), 2890. 10.3390/ijms26072890 40243470 PMC11988371

[B70] Massagué J. Sheppard D. (2023). TGF-β signaling in health and disease. Cell 186 (19), 4007–4037. 10.1016/j.cell.2023.07.036 37714133 PMC10772989

[B71] Mazumder S. Mitra G. T. Mukherjee U. K. Chakravarti S. Amiri F. Waliagha R. S. (2022). Integrating pharmacogenomics data-driven computational drug prediction with single-cell RNAseq to demonstrate the efficacy of a NAMPT inhibitor against aggressive, taxane-resistant, and stem-like cells in lethal prostate cancer. Cancers (Basel) 14 (23), 6009. 10.3390/cancers14236009 36497496 PMC9738762

[B72] Meng Y. Guo D. Lin L. Zhao H. Xu W. Luo S. (2024). Glycolytic enzyme PFKL governs lipolysis by promoting lipid droplet-mitochondria tethering to enhance β-oxidation and tumor cell proliferation. Nat. Metab. 6 (6), 1092–1107. 10.1038/s42255-024-01047-2 38773347

[B73] Miao L. Yang L. Li R. Rodrigues D. N. Crespo M. Hsieh J. T. (2017). Disrupting androgen receptor signaling induces snail-mediated epithelial-mesenchymal plasticity in prostate cancer. Cancer Res. 77 (11), 3101–3112. 10.1158/0008-5472.CAN-16-2169 28302679

[B74] Mickova A. Kharaishvili G. Kurfurstova D. Gachechiladze M. Kral M. Vacek O. (2021). Skp2 and Slug are coexpressed in aggressive prostate cancer and inhibited by neddylation blockade. Int. J. Mol. Sci. 22 (6), 2844. 10.3390/ijms22062844 33799604 PMC8000894

[B75] Mirzaei S. Paskeh M. D. A. Saghari Y. Zarrabi A. Hamblin M. R. Entezari M. (2022). Transforming growth factor-beta (TGF-β) in prostate cancer: a dual function mediator? Int. J. Biol. Macromol. 206, 435–452. 10.1016/j.ijbiomac.2022.02.094 35202639

[B76] Mitchell K. Sprowls S. A. Arora S. Shakya S. Silver D. J. Goins C. M. (2023). WDR5 represents a therapeutically exploitable target for cancer stem cells in glioblastoma. Genes Dev. 37 (3-4), 86–102. 10.1101/gad.349803.122 36732025 PMC10069451

[B77] Moll J. M. Teubel W. J. Erkens S. E. Jozefzoon-Agai A. Dits N. F. van Rijswijk A. (2022). Cell line characteristics predict subsequent resistance to androgen receptor-targeted agents (ARTA) in preclinical models of prostate cancer. Front. Oncol. 12, 877613. 10.3389/fonc.2022.877613 35769712 PMC9234122

[B78] Moyret-Lalle C. Prodhomme M. K. Burlet D. Kashiwagi A. Petrilli V. Puisieux A. (2022). Role of EMT in the DNA damage response, double-strand break repair pathway choice and its implications in cancer treatment. Cancer Sci. 113 (7), 2214–2223. 10.1111/cas.15389 35534984 PMC9277259

[B79] Navas T. Kinders R. J. Lawrence S. M. Ferry-Galow K. V. Borgel S. Hollingshead M. G. (2020). Clinical evolution of epithelial-mesenchymal transition in human carcinomas. Cancer Res. 80 (2), 304–318. 10.1158/0008-5472.CAN-18-3539 31732654 PMC8170833

[B80] Nguyen D. P. Li J. Tewari A. K. (2014). Inflammation and prostate cancer: the role of interleukin 6 (IL-6). BJU Int. 113 (6), 986–992. 10.1111/bju.12452 24053309

[B81] Ni J. Cozzi P. Hao J. Beretov J. Chang L. Duan W. (2013). Epithelial cell adhesion molecule (EpCAM) is associated with prostate cancer metastasis and chemo/radioresistance via the PI3K/Akt/mTOR signaling pathway. Int. J. Biochem. Cell Biol. 45 (12), 2736–2748. 10.1016/j.biocel.2013.09.008 24076216

[B82] Nikhil K. Kamra M. Raza A. Haymour H. S. Shah K. (2020). Molecular interplay between AURKA and SPOP dictates CRPC pathogenesis via androgen receptor. Cancers (Basel) 12 (11), 3247. 10.3390/cancers12113247 33158056 PMC7693105

[B83] Pal S. K. He M. Wilson T. Liu X. Zhang K. Carmichael C. (2015). Detection and phenotyping of circulating tumor cells in highrisk localized prostate cancer. Clin. Genitourin. Cancer 13 (2), 130–136. 10.1016/j.clgc.2014.08.014 25450039 PMC4479263

[B84] Paller C. Pu H. Begemann D. E. Wade C. A. Hensley P. J. Kyprianou N. (2019). TGF-β receptor I inhibitor enhances response to enzalutamide in a pre-clinical model of advanced prostate cancer. Prostate 79 (1), 31–43. 10.1002/pros.23708 30155899 PMC8444158

[B85] Pan G. Liu Y. Shang L. Zhou F. Yang S. (2021). EMT-associated microRNAs and their roles in cancer stemness and drug resistance. Cancer Commun. (Lond). 41 (3), 199–217. 10.1002/cac2.12138 33506604 PMC7968884

[B86] Parol M. Gzil A. Bodnar M. Grzanka D. (2021). Systematic review and meta-analysis of the prognostic significance of microRNAs related to metastatic and EMT process among prostate cancer patients. J. Transl. Med. 19 (1), 28. 10.1186/s12967-020-02644-x 33413466 PMC7788830

[B87] Pastushenko I. Blanpain C. (2019). EMT transition states during tumor progression and metastasis. Trends Cell Biol. 29 (3), 212–226. 10.1016/j.tcb.2018.12.001 30594349

[B88] Pitt J. M. Marabelle A. Eggermont A. Soria J. C. Kroemer G. Zitvogel L. (2016). Targeting the tumor microenvironment: removing obstruction to anticancer immune responses and immunotherapy. Ann. Oncol. 27 (8), 1482–1492. 10.1093/annonc/mdw168 27069014

[B89] Preisser F. Abrams-Pompe R. S. Stelwagen P. J. Böhmer D. Zattoni F. Magli A. (2024). European association of Urology biochemical recurrence risk classification as a decision tool for salvage radiotherapy-A multicenter study. Eur. Urol. 85 (2), 164–170. 10.1016/j.eururo.2023.05.038 37355358

[B90] Qu J. Li J. Zhang Y. He R. Liu X. Gong K. (2021). AKR1B10 promotes breast cancer cell proliferation and migration via the PI3K/AKT/NF-κB signaling pathway. Cell Biosci. 11 (1), 163. 10.1186/s13578-021-00677-3 34419144 PMC8379827

[B91] Rajamäki K. Taira A. Katainen R. Välimäki N. Kuosmanen A. Plaketti R. M. (2021). Genetic and epigenetic characteristics of inflammatory bowel disease-associated colorectal cancer. Gastroenterology 161 (2), 592–607. 10.1053/j.gastro.2021.04.042 33930428

[B92] Ruan D. He J. Li C. F. Lee H. J. Liu J. Lin H. K. (2017). Skp2 deficiency restricts the progression and stem cell features of castration-resistant prostate cancer by destabilizing Twist. Oncogene 36 (30), 4299–4310. 10.1038/onc.2017.64 28346424 PMC5532065

[B93] sai Y. C. Chen W. Y. Abou-Kheir W. Zeng T. Yin J. J. Bahmad H. (2018). Androgen deprivation therapy-induced epithelialmesenchymal transition of prostate cancer through downregulating SPDEF and activating CCL2. Biochim. Biophys. Acta Mol. Basis Dis. 1864 (5 Pt A), 1717–1727. 10.1016/j.bbadis.2018.02.016 29477409

[B94] Saitoh M. (2023). Transcriptional regulation of EMT transcription factors in cancer. Semin. Cancer Biol. 97, 21–29. 10.1016/j.semcancer.2023.10.001 37802266

[B95] Shen J. Wang M. Li F. Yan H. Wang R. Zhou J. (2022). Establishment and validation of a model for disease-free survival rate prediction using the combination of microRNA-381 and clinical indicators in patients with breast cancer. Breast Cancer (Dove Med. Press) 14, 375–389. 10.2147/BCTT.S383121 36475295 PMC9719690

[B96] Shu D. Y. Butcher E. Saint-Geniez M. (2020). EMT and EndMT: emerging roles in age-related macular degeneration. Int. J. Mol. Sci. 21 (12), 4271. 10.3390/ijms21124271 32560057 PMC7349630

[B97] Siegel R. L. Miller K. D. Wagle N. S. Jemal A. (2023). Cancer statistics, 2023. CA Cancer J. Clin. 73 (1), 17–48. 10.3322/caac.21763 36633525

[B98] Singh M. Yelle N. Venugopal C. Singh S. K. (2018). EMT: mechanisms and therapeutic implications. Pharmacol. Ther. 182, 80–94. 10.1016/j.pharmthera.2017.08.009 28834698

[B99] Song B. Park S. H. Zhao J. C. Fong K. W. Li S. Lee Y. (2019). Targeting FOXA1-mediated repression of TGF-β signaling suppresses castration-resistant prostate cancer progression. J. Clin. Invest 129 (2), 569–582. 10.1172/JCI122367 30511964 PMC6355239

[B100] Soundararajan R. Paranjape A. N. Maity S. Aparicio A. Mani S. A. (2018). EMT, stemness and tumor plasticity in aggressive variant neuroendocrine prostate cancers. Biochim. Biophys. Acta Rev. Cancer 1870 (2), 229–238. 10.1016/j.bbcan.2018.06.006 29981816 PMC6496942

[B101] Sun Y. Jing J. Xu H. Xu L. Hu H. Tang C. (2021). N-cadherin inhibitor creates a microenvironment that protect TILs from immune checkpoints and Treg cells. J. Immunother. Cancer 9 (3), e002138. 10.1136/jitc-2020-002138 33692219 PMC7949480

[B102] Sun Y. Wang B. E. Leong K. G. Yue P. Li L. Jhunjhunwala S. (2012). Androgen deprivation causes epithelial-mesenchymal transition in the prostate: implications for androgen-deprivation therapy. Cancer Res. 72 (2), 527–536. 10.1158/0008-5472.CAN-11-3004 22108827

[B103] Taki M. Abiko K. Ukita M. Murakami R. Yamanoi K. Yamaguchi K. (2021). Tumor immune microenvironment during epithelial-mesenchymal transition. Clin. Cancer Res. 27 (17), 4669–4679. 10.1158/1078-0432.CCR-20-4459 33827891

[B104] Tan J. Hu R. Gong J. Fang C. Li Y. Liu M. (2023). Protection against metabolic associated fatty liver disease by protocatechuic acid. Gut Microbes 15 (1), 2238959. 10.1080/19490976.2023.2238959 37505920 PMC10392757

[B105] Tan T. Shi P. Abbas M. N. Wang Y. Xu J. Chen Y. (2022). Epigenetic modification regulates tumor progression and metastasis through EMT (Review). Int. J. Oncol. 60 (6), 70. 10.3892/ijo.2022.5360 35445731 PMC9084613

[B106] Tanabe S. Quader S. Cabral H. Perkins E. J. Yokozaki H. Sasaki H. (2024). Master regulators of causal networks in intestinal- and diffuse-type gastric cancer and the relation to the RNA virus infection pathway. Int. J. Mol. Sci. 25 (16), 8821. 10.3390/ijms25168821 39201509 PMC11354771

[B107] Teng M. Zhou S. Cai C. Lupien M. He H. H. (2021). Pioneer of prostate cancer: past, present and the future of FOXA1. Protein Cell 12 (1), 29–38. 10.1007/s13238-020-00786-8 32946061 PMC7815845

[B108] Teo M. Y. Rathkopf D. E. Kantoff P. (2019). Treatment of advanced prostate cancer. Annu. Rev. Med. 70, 479–499. 10.1146/annurev-med-051517-011947 30691365 PMC6441973

[B109] Therachiyil L. Krishnankutty R. Ahmad F. Mateo J. M. Uddin S. Korashy H. M. (2022). Aryl hydrocarbon receptor promotes cell growth, stemness like characteristics, and metastasis in human ovarian cancer via activation of PI3K/Akt, β-catenin, and epithelial to mesenchymal transition pathways. Int. J. Mol. Sci. 23 (12), 6395. 10.3390/ijms23126395 35742838 PMC9223661

[B110] Torrealba N. Vera R. Fraile B. Martínez-Onsurbe P. Paniagua R. Royuela M. (2020). TGF-β/PI3K/AKT/mTOR/NF-kB pathway. Clinicopathological features in prostate cancer. Clin. Featur. prostate cancer. Aging Male 23 (5), 801–811. 10.1080/13685538.2019.1597840 30973040

[B111] Tsao C. W. Li J. S. Lin Y. W. Wu S. T. Cha T. L. Liu C. Y. (2021). Regulation of carcinogenesis and mediation through Wnt/β-catenin signaling by 3,3'-diindolylmethane in an enzalutamide-resistant prostate cancer cell line. Sci. Rep. 11 (1), 1239. 10.1038/s41598-020-80519-3 33441906 PMC7806813

[B112] Valastyan S. Weinberg R. A. (2011). Tumor metastasis: molecular insights and evolving paradigms. Cell 147 (2), 275–292. 10.1016/j.cell.2011.09.024 22000009 PMC3261217

[B113] Vasan N. Baselga J. Hyman D. M. (2019). A view on drug resistance in cancer. Nature 575 (7782), 299–309. 10.1038/s41586-019-1730-1 31723286 PMC8008476

[B114] Verma P. Shukla N. Kumari S. Ansari M. S. Gautam N. K. Patel G. K. (2023). Cancer stem cell in prostate cancer progression, metastasis and therapy resistance. Biochim. Biophys. Acta Rev. Cancer 1878 (3), 188887. 10.1016/j.bbcan.2023.188887 36997008

[B115] Wang F. Hu S. Ma D. Q. Li Q. Li H. C. Liang J. Y. (2022). ER/AR multi-conformational docking server: a tool for discovering and studying estrogen and androgen receptor modulators. Front. Pharmacol. 13, 800885. 10.3389/fphar.2022.800885 35140614 PMC8819068

[B116] Wang H. Fang R. Wang X. F. Zhang F. Chen D. Y. Zhou B. (2013). Stabilization of Snail through AKT/GSK-3β signaling pathway is required for TNF-α-induced epithelial-mesenchymal transition in prostate cancer PC3 cells. Eur. J. Pharmacol. 714 (1-3), 48–55. 10.1016/j.ejphar.2013.05.046 23769744

[B117] Wang H. Li N. Liu Q. Guo J. Pan Q. Cheng B. (2023). Antiandrogen treatment induces stromal cell reprogramming to promote castration resistance in prostate cancer. Cancer Cell 41 (7), 1345–1362.e9. 10.1016/j.ccell.2023.05.016 37352863

[B118] Wang M. Ren D. Guo W. Huang S. Wang Z. Li Q. (2016). N-cadherin promotes epithelial-mesenchymal transition and cancer stem cell-like traits via ErbB signaling in prostate cancer cells. Int. J. Oncol. 48 (2), 595–606. 10.3892/ijo.2015.3270 26647992

[B119] Wang P. Du S. Guo C. Ni Z. Huang Z. Deng N. (2024). The presence of blastocyst within the uteri facilitates lumenal epithelium transformation for implantation via upregulating lysosome proteostasis activity. Autophagy 20 (1), 58–75. 10.1080/15548627.2023.2247747 37584546 PMC10761037

[B120] Wang S. T. Wang Y. Y. Huang J. R. Shu Y. B. He K. Shi Z. (2024). THZ2 ameliorates mouse colitis and colitis-associated colorectal cancer. Biomedicines 12 (3), 679. 10.3390/biomedicines12030679 38540292 PMC10967821

[B121] Wang T. Jin J. Qian C. Lou J. Lin J. Xu A. (2021). Estrogen/ER in anti-tumor immunity regulation to tumor cell and tumor microenvironment. Cancer Cell Int. 21 (1), 295. 10.1186/s12935-021-02003-w 34098945 PMC8182917

[B122] Wang Y. Ping Z. Gao H. Liu Z. Xv Q. Jiang X. (2024). LYC inhibits the AKT signaling pathway to activate autophagy and ameliorate TGFB-induced renal fibrosis. Autophagy 20 (5), 1114–1133. 10.1080/15548627.2023.2287930 38037248 PMC11135866

[B123] Wei Z. Zhang C. Song Y. Han D. Liu J. Song X. (2024). CircUBE3A(2,3,4,5) promotes adenylate-uridylate-rich binding factor 1 nuclear translocation to suppress prostate cancer metastasis. Cancer Lett. 588, 216743. 10.1016/j.canlet.2024.216743 38423246

[B124] Wu C. Guo E. Ming J. Sun W. Nie X. Sun L. (2020). Radiation-induced DNMT3B promotes radioresistance in nasopharyngeal carcinoma through methylation of p53 and p21. Mol. Ther. Oncolytics 17, 306–319. 10.1016/j.omto.2020.04.007 32382655 PMC7200625

[B125] Yang C. Huang X. Liu Z. Qin W. Wang C. (2020). Metabolism-associated molecular classification of hepatocellular carcinoma. Mol. Oncol. 14 (4), 896–913. 10.1002/1878-0261.12639 31955511 PMC7138397

[B126] Yang J. Antin P. Berx G. Blanpain C. Brabletz T. Bronner M. (2020). Guidelines and definitions for research on epithelial–mesenchymal transition. Nat. Rev. Mol. Cell Biol. 21, 341–352. 10.1038/s41580-020-0237-9 32300252 PMC7250738

[B127] Yang J. Ren B. Ren J. Yang G. Fang Y. Wang X. (2023). Epigenetic reprogramming-induced guanidinoacetic acid synthesis promotes pancreatic cancer metastasis and transcription-activating histone modifications. J. Exp. Clin. Cancer Res. 42 (1), 155. 10.1186/s13046-023-02698-x 37370109 PMC10304235

[B128] Yang Q. Zhao S. Shi Z. Cao L. Liu J. Pan T. (2021). Chemotherapy-elicited exosomal miR-378a-3p and miR-378d promote breast cancer stemness and chemoresistance via the activation of EZH2/STAT3 signaling. J. Exp. Clin. Cancer Res. 40 (1), 120. 10.1186/s13046-021-01901-1 33823894 PMC8022546

[B129] Yu O. H. Y. Suissa S. (2023). Metformin and cancer: solutions to a real-world evidence failure. Diabetes Care 46 (5), 904–912. 10.2337/dci22-0047 37185680

[B130] Zhang P. Hu P. Shen H. Yu J. Liu Q. Du J. (2014). Prognostic role of Twist or Snail in various carcinomas: a systematic review and meta-analysis. Eur. J. Clin. Invest 44 (11), 1072–1094. 10.1111/eci.12343 25257753

[B131] Zhang Z. Bao C. Jiang L. Wang S. Wang K. Lu C. (2023). When cancer drug resistance meets metabolomics (bulk, single-cell and/or spatial): progress, potential, and perspective. Front. Oncol. 12, 1054233. 10.3389/fonc.2022.1054233 36686803 PMC9854130

[B132] Zhang Z. J. Huang Y. P. Liu Z. T. Wang Y. X. Zhou H. Hou K. X. (2023). Identification of immune related gene signature for predicting prognosis of cholangiocarcinoma patients. Front. Immunol. 14, 1028404. 10.3389/fimmu.2023.1028404 36817485 PMC9932535

[B133] Zhao X. Yang Y. Yu H. Wu W. Sun Y. Pan Y. (2020). Polydatin inhibits ZEB1-invoked epithelial-mesenchymal transition in fructose-induced liver fibrosis. J. Cell Mol. Med. 24 (22), 13208–13222. 10.1111/jcmm.15933 33058500 PMC7701525

[B134] Zheng Z. Li J. Liu Y. Shi Z. Xuan Z. Yang K. (2022). The crucial role of AR-V7 in enzalutamide-resistance of castration-resistant prostate cancer. Cancers (Basel) 14 (19), 4877. 10.3390/cancers14194877 36230800 PMC9563243

[B135] Zhou W. Yao Y. Scott A. J. Wilder-Romans K. Dresser J. J. Werner C. K. (2020). Purine metabolism regulates DNA repair and therapy resistance in glioblastoma. Nat. Commun. 11 (1), 3811. 10.1038/s41467-020-17512-x 32732914 PMC7393131

